# Disparities in parental awareness of children’s seasonal influenza vaccination recommendations and influencers of vaccination

**DOI:** 10.1371/journal.pone.0230425

**Published:** 2020-04-09

**Authors:** Jane Tuckerman, Nigel W. Crawford, Helen S. Marshall

**Affiliations:** 1 Discipline of Paediatrics, Adelaide Medical School, University of Adelaide, Adelaide, South Australia, Australia; 2 Robinson Research Institute, University of Adelaide, Adelaide, South Australia, Australia; 3 Department of Paediatrics, University of Melbourne & Murdoch Children’s Research Institute (MCRI), Melbourne, Australia; 4 Department of General Medicine, Royal Children's Hospital, Melbourne, Australia; 5 Vaccinology and Immunology Research Trials Unit, Women’s and Children’s Hospital, North Adelaide, South Australia, Australia; 6 South Australian Health and Medical Research Institute, Adelaide, South Australia, Australia; University of Campania, ITALY

## Abstract

**Objective:**

To determine parental awareness of influenza vaccination recommendations for children and explore associations with awareness.

**Design:**

Cross-sectional survey.

**Setting/participants:**

South Australian parents with a telephone listing in the Electronic White Pages were randomly selected.

**Methods:**

Participants were interviewed using Computer Assisted Telephone Interviewing (CATI) during May–July 2016. Univariable and multivariable analyses explored characteristics associated with awareness; with the survey data weighted to reflect the population of SA and the probability of selection within a household.

**Results:**

Of 539 parents, 33% were aware of the recommendation that all children (<5 years) should receive the influenza vaccine annually with 51.9% aware that children with special risk medical conditions (SRMC) should also receive the vaccine annually. Characteristics strongly associated with parental awareness of the recommendation for children aged < 5 years were knowledge of recommendation for children with a SRMC (adjusted Odds Ratio [aOR] 10.46, CI 4.44–24.63) or living in a metropolitan area (aOR 2.91, CI 1.19–7.09). There was lack of awareness in those not working (aOR 0.13, CI 0.04–0.47), with trade level education (compared with high school) (aOR 0.25 CI, 0.09–0.71) and in those born in the UK or Ireland (aOR 0.19, CI 0.04–0.85). Awareness of the recommendation for children with SRMC to receive the vaccine was strongly associated with knowledge of the influenza recommendation for children <5 years (aOR 10.22, CI 4.39–23.77) or not being born in Australia [UK/ Ireland (aOR 7.63, CI 1.86–31.31); other (aOR 3.93, CI 0.94–16.42)]. The most influential cues to future receipt were a general practitioner (GP) recommendation (63.8%) and providing influenza vaccine free for all children (37.6%). More parents who delayed or excluded vaccines believed that their children’s vaccinations (in general) were unnecessary, as other children were vaccinated (42.8%) compared to those with no or minor concerns (11.1%) (p<0.0001).

**Conclusions:**

Parental awareness of children’s influenza vaccine recommendations is low. Targeted communication strategies and resources are required to establish broader community awareness of recommendations. Healthcare provider endorsement of the vaccine remains key and health care professionals, particularly GPs and paediatric specialists should be encouraged to discuss influenza vaccine with parents at every opportunity. Many parents have vaccine concerns and addressing concerns across the spectrum of hesitancy is crucial.

## Introduction

Influenza is the leading cause of vaccine preventable hospitalisations for Australian children aged under 5 years. [1, 2] Children experience considerable disease burden with a higher annual incidence than adults. Ten to forty percent of children are infected each year, which increases considerably in children attending day-care. [3–5] Children also shed higher levels of the virus for a longer period, contributing to the virus’ circulation within the community.[6–8] Attributable healthcare costs of influenza in children are substantial, as are indirect economic losses including lost productivity through parents needing time off work to care for infected children and subsequent secondary transmission in households. [9–11]

**Fig 1:**
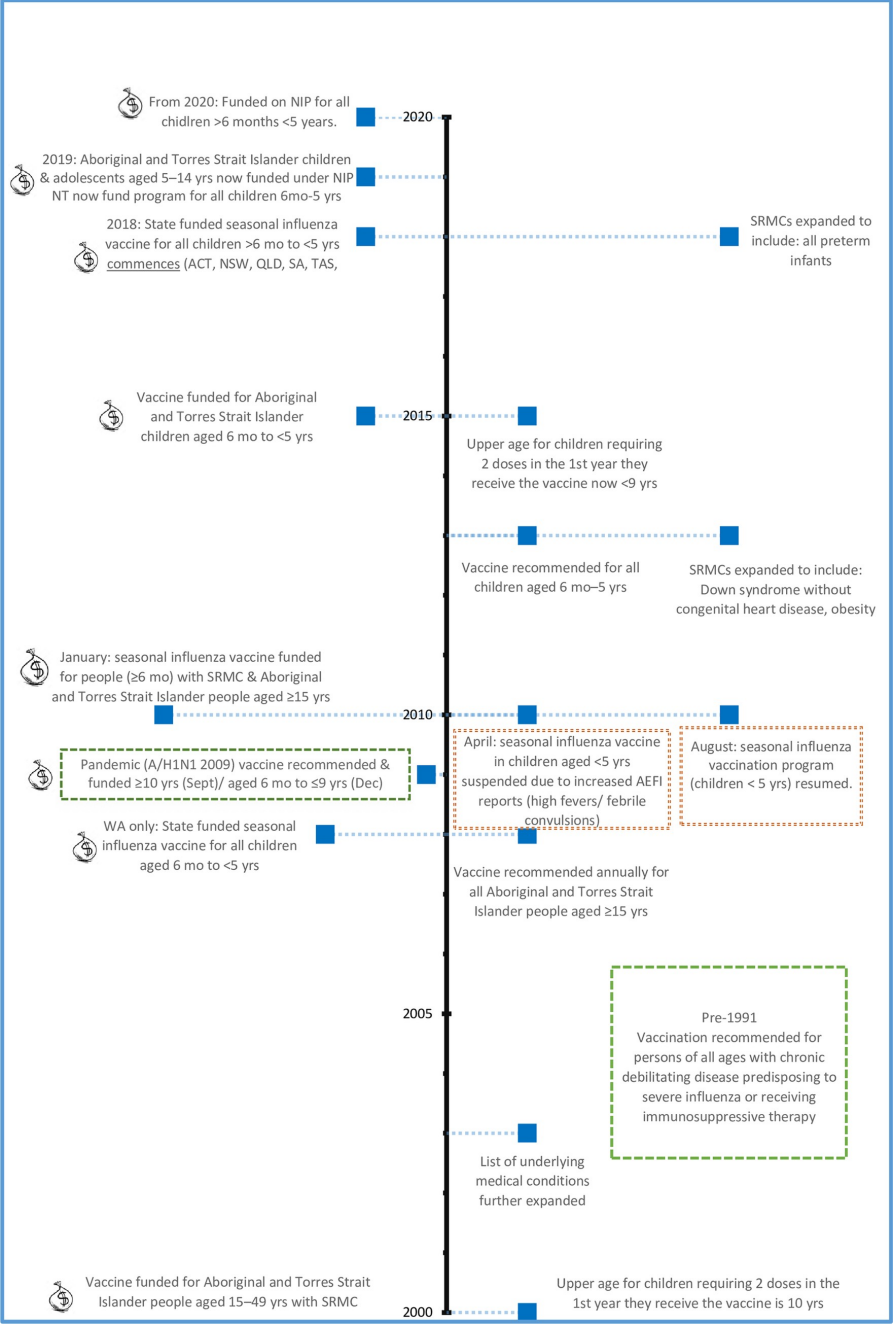
Brief timeline of children's seasonal influenza vaccination recommendations, funding and events in Australia.

In recent times, changes to recommendations, funding, the 2009/2010 pandemic and a cluster of serious adverse events with use of the BioCSL Fluvax vaccine have contributed to the changing landscape of children’s influenza vaccination in Australia (Fig 1). Influenza vaccine coverage in Australian children has been poor, with previous uptake reported between 14–23% in children aged <18 years [12–15]. More recent 2018 data reports coverage for children aged <5 years ranges between 19–43.4% across jurisdictions (25.6% overall). [16] Estimated coverage is 30–44% for children with special risk medical conditions (SRMC)[12, 17, 18].

Footnote: SRMC: Special Risk Medical Condition; mo: months; yrs: years. ACT: Australian Capital Territory; NSW: New South Wales; QLD: Queensland; SA: South Australia; TAS: Tasmania; VIC: Victoria; NT: Northern Territory; WA: Western Australia. Reference: National Centre for Immunisation Research and Surveillance (NCIRS). Significant events in influenza vaccination in Australia: NCIRS Fact sheet. Significant events in influenza vaccination in Australia: NCIRS Fact sheet April 2019. [19]

The influenza vaccine is free to those eligible under Australia’s National Seasonal Influenza Vaccination and at a cost to non-eligible people, with the vaccine accessible through medical practices, community immunisation clinics, hospitals, community children’s health clinics and Aboriginal Health Centres. Community pharmacies may also administer the vaccine at a cost, dependent on the jurisdiction and a person’s age.

While a doctor’s recommendation is considered central to influenza vaccine uptake in children [12, 17, 20–24], much of the previous research examining factors related to vaccine uptake in children in both Australia and elsewhere [12, 17, 20–23, 25–28] was undertaken in children hospitalised or with SRMC. However, it is likely that general parental awareness of children’s influenza recommendations also contributes to uptake and there is limited research on parental awareness from a community perspective.

Only 50% of parents in a recent Australian study knew the vaccine was recommended for children < 5 years, while another found higher uptake if parents believed the vaccine was recommended for their child’s age group or with the same medical condition as their child. [12, 13] Lack of parental awareness of the recommendation is cited as a common reason for non-vaccination in children with SRMC [29] and hospitalised children [20].

Understanding parental awareness towards recommendations is essential in planning and developing strategies to increase uptake. The primary aim of this study was to examine parental awareness of influenza vaccine recommendations and explore associated characteristics. The study also sought to describe influences towards future receipt of the influenza vaccine, examine patterns of information provision and decision making towards vaccination in general, from a random sample of parents residing in South Australia (SA).

## Methods

### Study design

This study used data collected as part of a cross-sectional telephone survey. Study findings are reported with consideration of the STROBE statement.[30]

### Study setting

The survey was performed as part of the ‘Health Monitor’ program administered by the Population Research and Outcomes Studies Unit, University of Adelaide, with the study population recruited from the approximate 765,786 households located in metropolitan and rural South Australia during May–July 2016.

### Study recruitment

For this study, South Australian adults aged >18 years who were the parent or caregiver, referred to hereafter as parents, of a child aged <18 years were eligible to participate. Participants were members of households randomly selected from the SA Electronic White Pages (EWP) telephone listings in SA. For each household, the adult aged 18 years or older with the most recent birthday was selected for an interview. Each individual parent interviewed represents a separate household. Selected persons were non-replaceable, and interviews were not conducted with alternative household members if the selected person was not available. Up to 10 call-backs were made to each household before the selected individual was classified as a non-contact.

Ethical approval was granted from The University of Adelaide, and the SA Health HREC. Potential participants were informed of the purpose of the survey and timeframe, its voluntary nature and that they could decline or refuse questions at any stage of the survey or withdraw completely at any time. Consent was implied by participation.

### Parental survey questionnaire

An independent external research company conducted Computer Assisted Telephone Interviews (CATI) whereby the interviewer followed a script provided by a computer and entered participant responses directly into a database. At the beginning of the dialogue, interviewers stated that they were calling on behalf of The University of Adelaide to conduct a survey on a range of health issues. There was an introductory sentence for each health topic covered in the survey and specific questions could be directed towards subgroups, such as parents. All data collected were non-identifiable. A pilot study of 50 randomly selected households tested question format and sequence. The questionnaire was designed so that each interview took on average 15 minutes or less to be completed.

Respondents who identified as a parent (of a child aged <18 years) were asked their awareness of children’s influenza vaccination recommendations and influencing factors towards future vaccine receipt for their child. Possible responses to future intentions towards influenza immunisation and immunisation service use were read to participants with the option for multiple response. Respondents could also specify another response, that was later recoded. To examine parental attitudes towards vaccines in general, parents were asked to state their beliefs towards vaccine necessity, side effects, access to services, behaviour towards their child receiving vaccines and their level of concern according to the Vaccine Communication Framework (VCF). [31] To examine immunisation service use, parents were asked their immunisation provider type, decision-making surrounding their choice as well as any difficulties with access. Parents were also asked their views on information surrounding where to obtain vaccinations and to rate their child’s most recent vaccination service. Parents were instructed to answer all immunisation specific questions in relation to their youngest child.

### Statistical analysis

The survey data were weighted by the inverse of the individual's probability of selection and the number of times their telephone number(s) is(are) listed in the EWP, then re-weighted to age group by sex by section of state (metropolitan/country) benchmarks derived from the June 2014 ABS Estimated Resident Population. Weighting corrected the distributions in the sample data to approximate those of the SA population. The weights generated for the wider study population were then maintained for the parental subset. Both as an expansion of the data and as a matter of adjustment for non-response and non-coverage, resulting in data that is representative of the population rather than limited to the households that responded.

Additional response questions were coded using content analysis and grouped into categories. Characteristics associated with awareness of the current influenza recommendations for children were explored in successive multivariable logistic regression models. All the variables were included and grouped together in blocks, facilitating an understanding of each group of variables role in explaining awareness.

These variable blocks representing different constructs, may be important in understanding vaccination awareness. The explanatory variable blocks were demographic variables, parental beliefs/attitudes and health service use/awareness of other influenza recommendations. Unadjusted odds ratios (OR) and adjusted OR (aOR) were presented with their 95% confidence intervals (CI). In this paper, the crude versus final model is presented, with S1 and S2 Tables showing full models. We examined level of parental concern according to the VCF. To further explore this, we summed the total number of questions each participant had responded to, in a negative or opposing way to 4 other questions related to vaccination beliefs and safety. Those who responded in the neutral category were not included (neither agree/disagree) as an expression of hesitancy. We used Stata (Version 14.1) for all statistical analyses (StataCorp, Texas, USA).

## Results

### Study population

From 5,200 households randomly selected to participate, 2,118 households could not be contacted or were non-residential telephone numbers. From the remaining 3,082 telephone numbers, 2,006 interviews were conducted, a participation rate of 64.8%. After the raw data were weighted, 547 (27.3%) participants were parents, with 539 providing complete data.

### Description of study sample

In the weighted sample, the mean age of parents was 41.5 years (95% CI 40.0–42.9) (Table 1). There were slightly more female parents (53.3%), with the majority Australian born (n = 436; 80.9%) and speaking English as the predominant household language (91.9%, n = 496). Most households were situated in metropolitan Adelaide (77.5%, n = 418) while 22.5% (n = 121) were rural/regional residences, closely reflecting the proportion of households in metropolitan versus rural South Australia.

**Table 1:** Household demographics of survey participants.

Participant characteristic		Eligible parents^#^	Parents^#^ with complete data †	
		Raw N = 285	Raw N = 279	Weighted (N = 539)
	Level	n (%)	n%	n%
Age (years)	25–34	27 (9.5)	26 (9.3)	121 (22.5)
	35–44	102 (35.8)	101 (36.2)	236 (43.8)
	45–54	117 (41.1)	115 (41.2)	152 (28.2)
	55 and over	39 (13.7)	37 (13.3)	30 (5.6)
Gender	Male	105 (36.8)	105 (37.6)	252 (46.7)
	Female	180 (63.2)	174 (62.4)	288 (53.3)
Residence	Regional	94 (33.0)	93 (33.3)	121 (22.5)
	Metropolitan	191 (67.0)	186 (66.7)	418 (77.5)
Country of Birth	Australia	238 (83.5)	234 (83.9)	436 (80.9)
	U.K. / Ireland	18 (6.3)	18 (6.5)	33 (6.1)
	Other	29 (10.2)	27 (9.7)	70 (13.1)
Main language in household	English	276 (96.8)	270 (96.8)	496 (91.9)
	Non-English	9 (3.2)	9 (3.2)	44 (8.1)
Educational attainment	High School or less	75 (26.3)	72 (25.8)	148 (27.4)
	Trade Certificate	101 (35.4)	99 (35.5)	173 (32.0)
	Bachelor or higher	109 (38.3)	108 (38.7)	219 (40.6)
Employment	Full time	137 (48.1)	135 (48.4)	292 (54.2)
	Part time/casual	99 (34.7)	99 (35.5)	166 (30.8)
	Not working	49 (17.2)	45 (16.1)	82 (15.1)
Household income	Up to $30,000	19 (6.7)	18 (6.5)	36 (6.7)
	$30,001 - $50,000	36 (12.6)	34 (12.2)	48 (8.9)
	$50,001 - $80,000	41 (14.4)	40 (14.3)	90 (16.6)
	$80,001 - $100,000	44 (15.4)	44 (15.8)	75 (13.9)
	More than $100,000	116 (40.7)	115 (41.2)	231 (42.8)
	Don't know/not stated	29 (10.2)	28 (10.0)	60 (11.1)

weighting can result in minor rounding variations.

#Participants who indicated they were the parent or caregiver of a child under the age of 18 years.

† We removed participants listwise with missing data for any of the variables included in the analysis (raw data n = 6).

### Awareness of seasonal influenza vaccination recommendations for children and characteristics associated with awareness

In total, 32.8% (n = 177) of parents were aware that all children aged >6months to 5 years are recommended to receive the influenza vaccine whereas 51.4% of parents (n = 277) were aware of the recommendation that children with SRMCs should receive the influenza vaccine. Only 26% (n = 141) of parents were aware of both recommendations. The proportion of parents who were aware of the recommendation for children with a SRMC to receive the vaccine was higher in parents who were aware of the recommendation towards all children < 5 years (140/177, 79.1%) compared with parents who were not aware of the recommendation for children < 5 years (136/362, 37.6%) (p<0.0001).

In the fully adjusted models (Table 2), awareness of the recommendation for children <5 years to receive the vaccine was strongly associated with knowledge of the influenza recommendation for children with a SRMC (aOR 10.46, CI 4.44–24.63) or living in a metropolitan area (aOR 2.91, CI 1.19–7.09) (Table 2). The model also indicated a lack of awareness in those not working (aOR 0.13, CI 0.04–0.47), with trade level education (compared with high school) (aOR 0.25 CI, 0.09–0.71) and in those born in the UK or Ireland (aOR 0.19, CI 0.04–0.85). Whilst awareness of the recommendation for children with SRMC to receive the vaccine was strongly associated with knowledge of the influenza recommendation for children <5 years (aOR 10.22, CI 4.39–23.77) or not being born in Australia [UK/ Ireland (aOR 7.63, CI 1.86–31.31); other (aOR 3.93, CI 0.94–16.42)]. The model also indicated awareness in female participants (aOR 2.47, CI 0.97–6.31).

**Table 2:** Multivariable results for the effect of characteristics on awareness of the influenza vaccine recommendations for children aged < 5 years and children with SRMC (N = 539).

				Awareness of the influenza vaccine recommendations for children aged < 5 years						Awareness of the influenza vaccine recommendations for children with SRMC					
Characteristic		Level	Number of parents	crude			Adjusted Model all covariates			crude			Adjusted Model all covariates		
				OR	95% CI	p value	OR	95% CI	p value	OR	95% CI	p value	OR	95% CI	p value
Demographic	Age (yrs)	-	539	0.99	(0.94–1.03)	0.562	0.99	(0.94–1.05)	0.787	1.01	(0.97–1.05)	0.513	1.02	(0.97–1.06)	0.460
	Gender	Female	288	0.98	(0.48–2.01)	0.957	1.00	(0.38–2.62)	0.998	1.78	(0.95–3.34)	0.071	2.47	(0.97–6.31)	0.058
	Residence location	Metropolitan (versus Regional)	418	2.03	(1.01–4.10)	0.047	2.91	(1.19–7.09)	0.019	1.12	(0.60–2.10)	0.712	0.77	(0.34–1.76)	0.535
	Country of birth	Australia	436	ref	-	-	ref	-	-	ref	-	-	ref	-	-
		UK/Ireland	33	0.27	(0.07–1.10)	0.067	0.19	(0.04–0.85)	0.030	3.72	(1.03–13.48)	0.046	7.63	(1.86–31.31)	0.005
		Other	70	1.36	(0.47–3.91)	0.566	0.48	(0.12–1.99)	0.313	1.48	(0.51–4.28)	0.469	3.93	(0.94–16.42)	0.060
	Household speaking language	Non-English (versus English)	44	1.64	(0.39–7.00)	0.502	2.83	(0.48–16.61)	0.250	0.85	(0.20–3.57)	0.827	0.28	(0.04–1.89)	0.190
	Highest educational level	High school or less	148	ref	-	-	ref	-	-	ref	-	-	ref	-	-
		Trade Certificate	173	0.29	(0.12–0.73)	0.008	0.25	(0.09–0.71)	0.010	0.88	(0.38–2.03)	0.770	1.64	(0.69–3.89)	0.266
		Bachelor or higher	219	0.64	(0.26–1.55)	0.321	0.55	(0.21–1.45)	0.230	0.77	(0.33–1.77)	0.535	0.89	(0.36–2.19)	0.808
	Employment type	Full time	292	ref	-	-	ref	-	-	ref	-	-	ref	-	-
		Part time/casual	166	1.02	(0.45–2.31)	0.958	0.63	(0.26–1.56)	0.319	1.65	(0.82–3.34)	0.162	1.37	(0.59–3.19)	0.460
		Not working	82	0.16	(0.06–0.42)	<0.001	0.13	(0.04–0.47)	0.002	0.89	(0.36–2.18)	0.797	0.85	(0.31–2.33)	0.748
Parental attitudes to immunisation	Vaccines are necessary to protect my children	Disagree*	9	ref	-	-	ref	-	-	ref	-	-	ref	-	-
		Neutral	29	0.56	(0.07–4.51)	0.588	1.00	(0.11–9.14)	0.997	1.40	(0.14–13.98)	0.775	1.13	(0.12–10.88)	0.917
		Agree**	502	0.45	(0.13–1.57)	0.211	0.51	(0.14–1.85)	0.305	0.92	(0.27–3.16)	0.895	0.63	(0.15–2.66)	0.531
	Belief that "immunisation is important to my everyday life"	No/ low importance^#^	15	ref	-	-	ref	-	-	ref	-	-	ref	-	-
		Neutral	13	0.35	(0.03–3.75)	0.388	1.62	(0.08–31.57)	0.749	0.71	(0.07–7.52)	0.773	0.15	(0.01–2.30)	0.173
		Important^##^	511	0.58	(0.09–3.82)	0.568	1.10	(0.13–9.45)	0.930	1.28	(0.19–8.40)	0.798	0.88	(0.09–8.77)	0.912
Health service use	Immunisation service provider	GP	355	ref	-	-	ref	-	-	ref	-	-	ref	-	-
		Community clinic	44	0.29	(0.09–0.94)	0.040	0.27	(0.05–1.48)	0.132	0.65	(0.24–1.79)	0.408	0.85	(0.18–4.10)	0.843
		Child health clinic	23	0.96	(0.19–4.92)	0.960	0.47	(0.09–2.61)	0.389	2.37	(0.66–8.56)	0.187	2.55	(0.77–8.48)	0.128
		Combination†	85	0.70	(0.26–1.86)	0.471	0.44	(0.18–1.06)	0.067	1.20	(0.52–2.78)	0.669	1.48	(0.62–3.51)	0.373
		Other††	23	0.53	(0.10–2.78)	0.450	0.19	(0.05–0.80)	0.024	4.32	(1.24–15.06)	0.022	13.40	(2.93–61.23)	0.001
		Don't vaccinate	9	1.29	(0.21–8.08)	0.782	0.92	(0.12–7.05)	0.933	0.76	(0.12–4.67)	0.763	0.23	(0.02–2.23)	0.203
	Youngest child has SRMC	Yes	26	0.48	(0.13–1.77)	0.271	0.95	(0.20–4.59)	0.949	0.92	(0.26–3.27)	0.899	0.74	(0.15–3.65)	0.713
Awareness other recommendations	Aware of influenza recommendation for children with SRMC	Yes	277	6.421	(2.73–15.11)	<0.001	10.46	(4.44–24.63)	<0.001						
	Aware of influenza recommendation for children <5 years	Yes	177							6.42	(2.73–15.11)	<0.001	10.22	(4.39–23.77)	<0.001

GP: general practitioner; SRMC: Special Risk Medical Conditions; Disagree

* included disagree/ strongly disagree; Agree

** included agree/ strongly agree; No/ low importance

# included responses ‘Not at all/ somewhat important’

## included Important/ Very important

^**†**^ included a combination of providers (from MP or clinics)

^**††**^other were school (n = 9), hospital (n = 4), chemist (n = 4), Aboriginal Health Service (n = 4) and 'Could not recall' (n = 2).

### Future influenza vaccination cues to action

**Fig 2:**
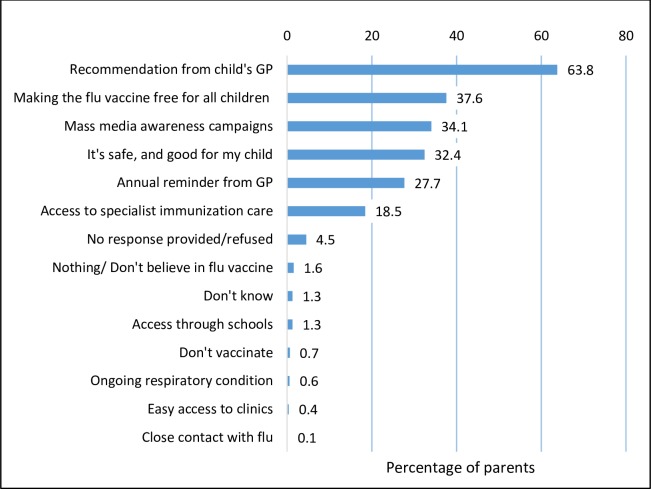
Potential influences towards future influenza vaccine receipt (N = 539).

Parents indicated a GP recommendation as the most influential cue to future influenza vaccination receipt (63.8%, n = 344) (Fig 2). This was followed by access to the vaccine at no cost for all children (37.6%, n = 203), greater awareness through mass media (34.1%, n = 184) with a third (32.4%, n = 175) citing the belief in the benefit of the vaccine as a key influence. Excluding parents who did not vaccinate altogether(n = 9), 41.6% (221/530) vaccinating parents were in favour of their child receiving the influenza vaccine from a pharmacy in the future, 48.3% were opposed and 10% undecided. Higher support for pharmacy provision came from parents in regional areas (48.8% versus 39.7% metropolitan), those working full (45%) or part time (45.7%) compared with those not working (21.7%) and in parents with lower educational attainment (high school (47.7%), trade 42.2% or bachelor (37.2%)).

Footnote: "What would be most influential for you in deciding to have your child receive a flu vaccine?" Multiple response—numbers will not total. GP: general practitioner.

### Vaccination in general

#### Decision-making

**Fig 3:**
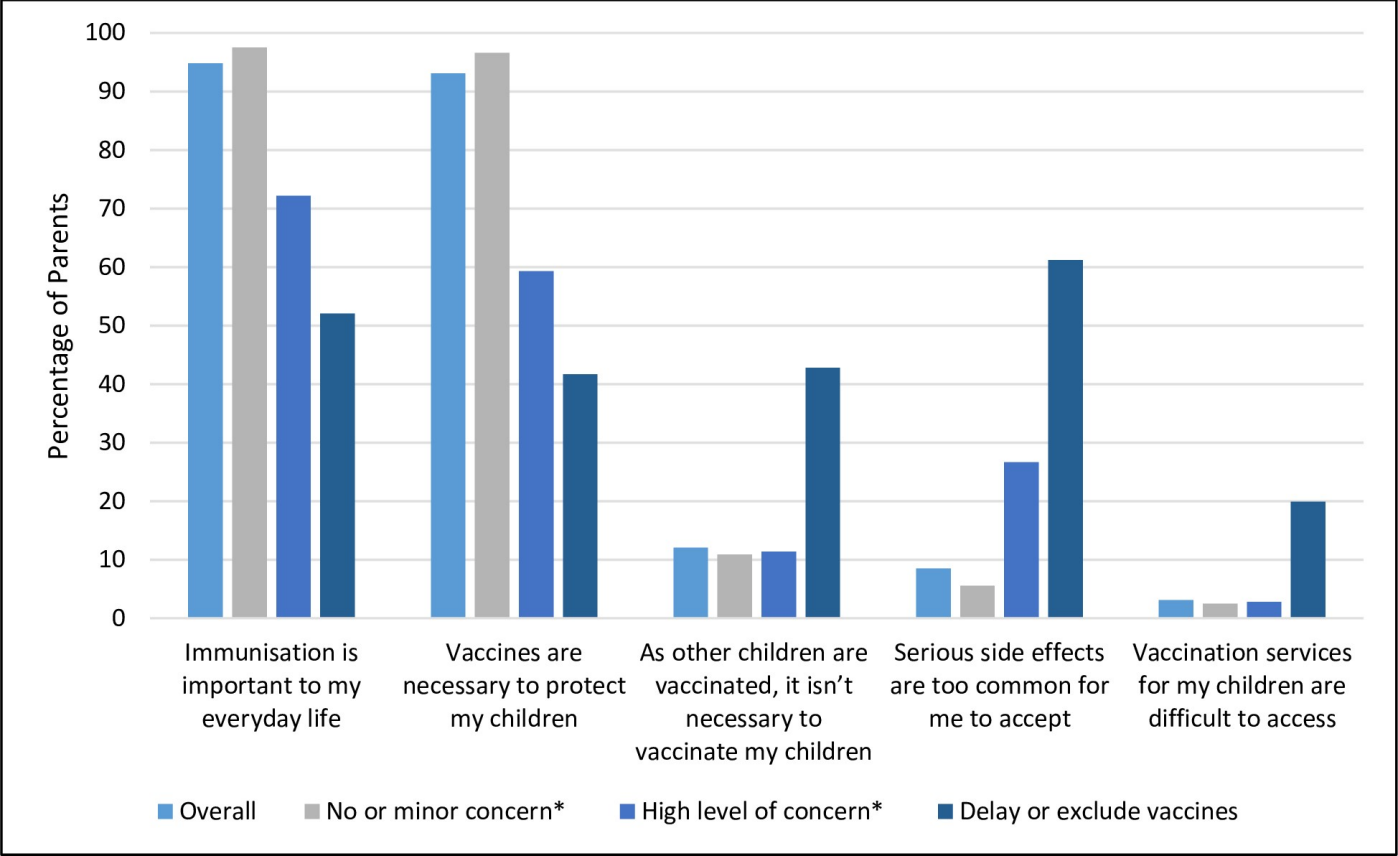
Parental agreement towards vaccination beliefs by level of concern towards vaccination (N = 539). * parent has vaccination concerns, but child receives all vaccines.

Overall, 23.8% expressed either minor (19.8%) or high (4.0%) concern towards vaccination in general yet still vaccinated; 1.9% delayed or excluded vaccines and 1.7% did not vaccinate at all. While 72.6% of parents reported having no concerns towards vaccination 15.2% (59/391) of this group’s responses to other vaccination belief questions indicated views opposing vaccination (S1 Fig). Of those reporting no concerns, 3.6% believed that vaccination is unnecessary to protect children, 10.2% thought vaccination was unnecessary as others are vaccinated, while 4.5% of this group held safety concerns. There were significant differences for specific vaccination beliefs based on a parent’s level of concern and behaviour towards vaccination (Fig 3). A higher proportion of parents who had no or minor concerns towards vaccination agreed/ strongly agreed that vaccines were necessary to protect their children (96.6%) compared to parents who reported a high level of concern (59.3%) or who delayed or excluded vaccines (41.7%) (p <0.0001) (S3 Table). Of concern, a higher proportion of parents who delayed or excluded vaccines reported agreeing /strongly agreeing that their children’s vaccinations were unnecessary as other children were vaccinated (42.8%) compared to those with no or minor concerns (10.9%) (p<0.0001). Over half of parents who delayed or excluded vaccines agreed/ strongly agreed that serious side effects were too common to accept (61.2%) compared to those with no or minor concerns (5.6%) (p<0.0001). A higher proportion of parents who delayed or excluded vaccines agreed/ strongly agreed that vaccination services were difficult to access (19.9%) compared to parents who reported a high level of concern (2.8%) or who had no or minor concerns (2.5%) (p = 0.005).

#### Immunisation service use (n = 530)

**Fig 4:**
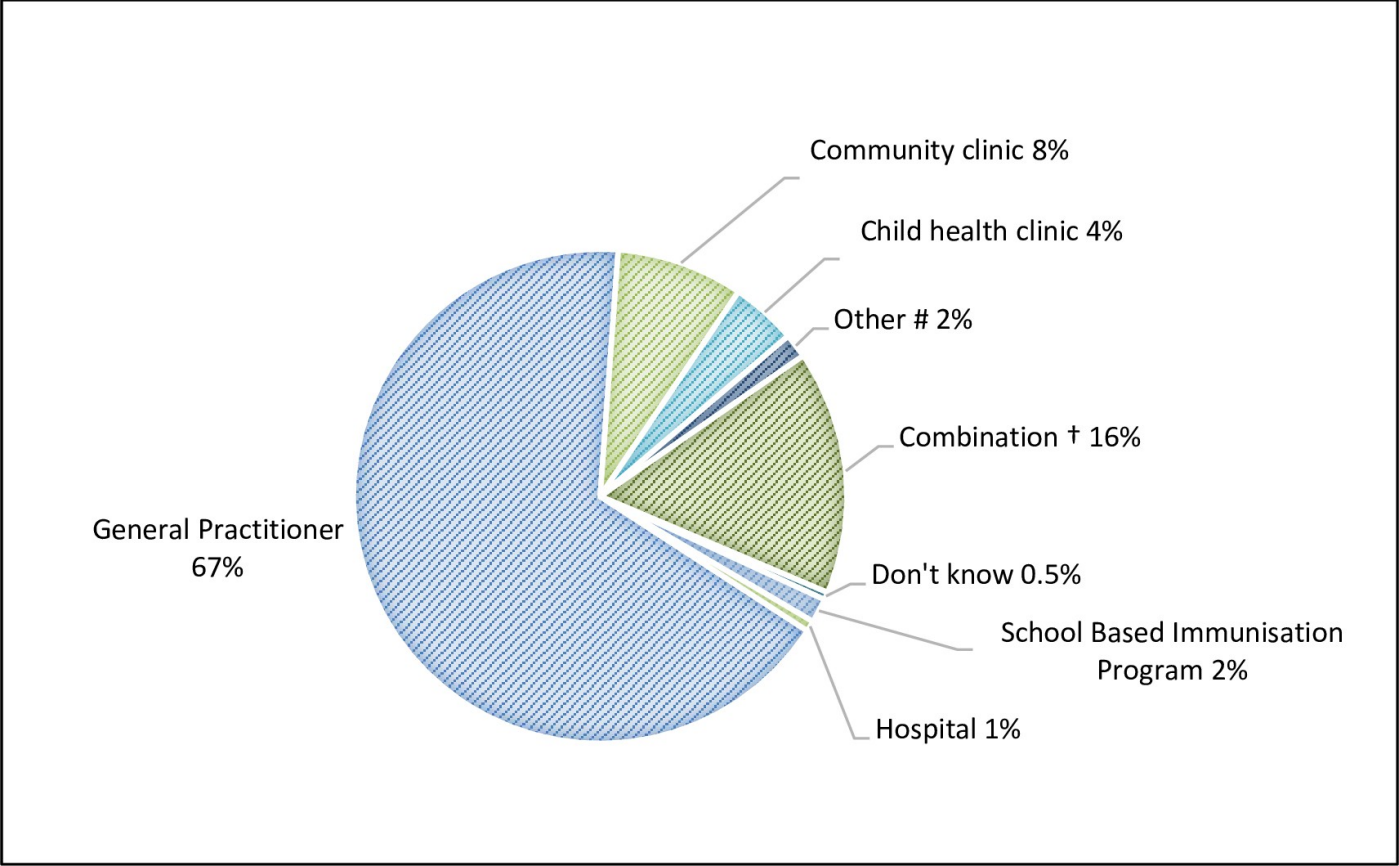
Children's immunisation providers (N = 530). These data exclude parents who did not vaccinate (n = 9; weighted data). Parents could only nominate one place. #: other were vaccinations administered by Aboriginal Health Service home visit (n = 4) and pharmacy (n = 4); † any combination of either family medical practitioner, community clinic or child health clinic.

All parents except those (n = 9) who indicated that their child did not receive any vaccinations provided information on their child’s most recent immunisation service. Children’s immunisations were received from their general practitioner (GP) (67%, n = 355.3), community immunisation clinic (8.4%, n = 44), child health clinic (4.3%, n = 23) or combination of clinics (16.2%) (Fig 4). Choice of immunisation provider was driven by proximity of the service to their home or easy access to (47.4%, n = 251) (S4 Table). Additionally, parents cited the immunisation service having their medical records (30.3%), being trustworthy (25.7%) or that a medical doctor provided the service (16.2%) as a reason for their choice of provider.

#### Cost and satisfaction with service

A small percentage of vaccinating parents (n = 72, 13.6%) recalled paying for the service to see their immunisation provider, such as medical practice, to obtain their child’s most recent vaccination, with a further 8% (n = 42) unable to recall. There was high satisfaction with the experience with 93.8% rating it as good or excellent and less than two percent of vaccinating parents (1.6%, n = 8) rating their child’s most recent vaccination service as fair or poor.

#### Sources of information on places to receive immunisations

A quarter (25.7%, n = 137) of vaccinating parents thought there was insufficient information on where they could obtain a vaccination for their child, with a further 6.9% undecided. Parents obtained information on the location of immunisation services from a variety of sources (S2 Fig). The major sources were medical practice (53.1%), news media (newspaper, radio or internet) (20%) and friends or other parents (12%).

## Discussion

For parents, vaccination decision making on behalf of their children can be a complex process. Awareness of the schedule and current recommendations forms a considerable part of this process and a cue to further action. Our study found only low to moderate awareness towards influenza vaccine recommendations for children.

Our finding of modest parental awareness of the influenza vaccination recommendation for children <5 years is lower than another Australian study [13] where 20% of parents incorrectly believed it is not recommended in this age group, with another 30% unsure. This Australian study reports the level of parent’s awareness towards the influenza vaccine recommendations for children from a community sample. Although, a recent study also found moderate awareness of the recommendation in parents of children with SRMCs [17], a much higher proportion of those who were aware of the recommendation were likely to receive it, even in the absence of a healthcare professional’s recommendation.

In our study, the strongest characteristic associated with awareness of the recommendation for children <5 was awareness of the recommendation for children with SRMC. However, given the change in effect size from the crude to final model, it is likely that some of the effect of ‘awareness of the SRMC recommendation’ was influenced by other variables. Being born in the UK/Ireland was also associated with lack of awareness with the difference in effect size between the crude and final adjusted model indicating little influence from other variables in the model. We also found a positive effect for residing in a metropolitan area that remained consistent, with little fluctuation in OR from the crude analysis to the final adjusted model indicating little influence from other variables in the model. The reason why residing in a metropolitan area showed such a strong effect is unclear. Research has previously identified regional GPs (non-metro) to be less likely to discuss non-funded immunisations. [32] While it is possible that this may indicate variability in access to GPs and medical care or an inadequate approach to influenza campaign messaging in general for regional areas, it may also be reflective of the health promotion messages and health education in regional areas more generally. The negative effect of not being employed also remained constant as did having trade level qualifications. It is possible that the type and amount of workforce participation mediate exposure to vaccination messages in general.

Our model exploring awareness of the recommendation for children with SRMC revealed the strongest characteristics associated with awareness was awareness of the recommendation for children <5 and being born outside Australia. Being a female participant was also associated with awareness of this recommendation and could reflect the fact that mothers are more likely to the primary carer of children with SRMC. The contrasting awareness towards the recommendations based on country of birth is interesting and possibly reflects policies towards recommendations in other countries. To further examine this and to test for the effect between ‘Aware of influenza recommendation…’ and other model variables, we included the related awareness variable last in each of the models and found it had only slight effect on other variables. For awareness of the recommendation (children < 5 years) the significance of immunisation provider diminished. It is possible that this had more to do with smaller numbers receiving vaccines at a community clinic (compared to GP) whilst influence of country of birth increased, with reduction in the boundaries and point estimate for those from UK/Ireland. Whilst for awareness of the recommendation for children with SRMC, the inverse is true with parents from Australia less likely to be aware of this recommendation.

### Immunisation service use

Our study showed a similar pattern of immunisation service use to that reported previously [33] with 66.7–82.9% of our sample receiving children’s vaccinations from their family GP. Our finding that the most influential factor for a child’s future influenza vaccination, nominated by parents, was a GP recommendation, is consistent with previous research that found a key facilitator for hospitalised children or children with SRMC was healthcare provider (HCP) recommendation. [12, 17, 20–24, 34] However, little has focused specifically on the cues to action towards influenza vaccination for children in general.

### Influenza vaccine access

Our study identified that access to vaccines may be problematic for particular groups of parents. More specifically, we found that parents who delayed or excluded vaccines reported finding vaccination services difficult to access. Additionally, that two in five parents would be willing for their child to receive the influenza vaccine from a pharmacy is also worth highlighting. Although Australian pharmacists began administering the influenza vaccine from 2014 (with variation across jurisdictions), only some states have recently endorsed delivery to children as young as 10 years of age. [35, 36] In contrast, pharmacists in several countries including Argentina, the United Kingdom (UK), Canada, Portugal, New Zealand and the USA (United States of America) have been administering the influenza vaccine for almost a decade. [37–39] With Canada, Argentina, the UK and several states in the USA also endorsing pharmacist administration to young children, with deviation in the minimum age requirement across countries. [37–39] In order to improve access, alternative delivery sites should be given consideration. Although generally Australian children do not start schooling until 5 years of age, one delivery option could be school-based influenza vaccination programs, such as those implemented in the UK and the USA [40, 41], delivery in childcare or pre-school programs or broader access through community clinics at extended times which would improve access overall, particularly during the peak influenza vaccination season.

### Community awareness of influenza vaccine

It is important to place community awareness within the context of recent Australian influenza vaccination events (Fig 1). In South Australia, at the time of this study the influenza vaccine was recommended but not funded for all children aged >6 months to < 5 years, although will be funded from 2020 as part of the NIP. [19] Our finding that a key motivator for 38.1% of parents was access to the vaccine at no cost is lower than other Australian studies reporting 48–55% of parents would be motivated by a free annual vaccine [13, 42] and this variance may be attributable to between study differences in the way data were obtained. These same studies also report stronger parental support towards a free influenza vaccine, than the actual cost being a financial barrier. [13, 42] The implications of a recommended but non-funded vaccine are well documented, with increased parental support for vaccines included on the NIP. [42, 43] This is also true of providers, with enhanced support for government funded vaccines and lower perceptions of disease severity towards non-funded vaccines previously noted.[32] Government funding implies to parents and providers alike that the vaccine is a priority, thereby influencing the decision-making process. Consideration of the implications of vaccine funding is therefore essential in the context of planning vaccination programs.

### Parental vaccine concerns

A third of parents (32.8%) reported belief in the vaccine’s safety and being beneficial to the child as a motivator to future vaccine receipt. Concerns about vaccine safety has remained a barrier to influenza vaccination in children complicated by the serious adverse events in 2010. [43, 44] Yet even prior to this, parental belief in the vaccine’s safety was shown to be associated with support for vaccination. [42] More recent studies have also found belief in the vaccine’s safety to be positively associated with vaccine uptake [12] but also suggest that negative publicity has lingered, complicated by parental knowledge towards the vaccine itself with a perception that the vaccine has not been around long enough, qualified by ensuring the safety of the vaccine. [45] Current data is encouraging however, as despite a 2017 poll indicating just 12% of parents believed the vaccine was safe this increased to 61% when the same poll was undertaken in 2018.[13, 46] This may have been influenced by 2017 being a high burden influenza season, with a number of deaths reported throughout Australia. [47]

Despite high recognition as to the importance of vaccines among parents in our study, a considerable number had vaccine concerns according to the Vaccine Communication Framework.[31] Parents who delayed or excluded vaccines placed lower importance on immunisation and doubted the necessity of vaccination to protect children. Worryingly, comparable proportions of parents who were highly concerned towards vaccinations also held these beliefs. Also consistent with recent literature were vaccine safety concerns [48–51] however, the high number of parents citing ‘free-riding’ logic as a reason not to vaccinate is also of concern and is higher than other recent Australian studies [15, 48, 49] and may indicate a need to address both the importance and benefits of vaccinations for these parents. The fact that parents who reportedly had no hesitations to vaccinate answered one or more questions about vaccination beliefs in an opposing way suggests that decision-making is complex and integrates many external factors. Understanding all the barriers to vaccination is important and while scales to determine the extent of parental hesitancy exist [52] establishing a tool on which to measure and categorise the broader spectrum of hesitancy is needed. Addressing parent’s vaccination concerns especially among those who are more hesitant is critical and requires an understanding of individual concerns, and provider communication, as highlighted previously [53], remains a key determinant of vaccine hesitancy. Moreover, GPs as primary health care providers play a key role in the immunisation landscape and have a pivotal role in providing recommendations for key targeted groups and new vaccines. [54–56] Given the high engagement of GPs in vaccination delivery, they remain in a key position of influence to support vaccine decision making, and communication resources such as SKAI—Sharing Knowledge About Immunisation [57] have been developed to assist in this process.

Previously much attention has focused on influenza recommendations for the elderly and the adult SRMC population with little attention paid to the awareness of influenza vaccination for children with SRMCs and children in general. The success of public health interventions rests of the swell of community attitudes towards such interventions and policies, the balance of perceived threat and risk of the intervention. Prior to this stage, however the community needs to be aware that a potential problem exists and be supplied with cues to action. A lack of awareness on the part of parents or HCPs may translate to missed opportunities for influenza decision making. Social norms may also influence community awareness, which has previously been highlighted in relation to children’s’ influenza vaccination [25, 42] and may warrant further examination.

The strength of this study is the unique perspective on awareness of influenza vaccine recommendations, with participants randomly sampled with weighting applied to improve the generalisability of the data. As a cross-sectional study however, it has some limitations. Although the study was conducted prior to implementation of state funded vaccine programs in Australia, our findings are still relevant to improving uptake of funded programs and have relevance to countries that recommend but do not fund influenza vaccine. Our sample was limited to those who spoke English and therefore the sample of non-English speaking parents was small. This is relevant considering that ethnicity has been identified as a significant factor in vaccination status [58, 59], with not speaking a country’s dominant language previously highlighted as a barrier for influenza vaccination. [60, 61] Accordingly, eliciting the views of non-English-speaking households is important to improving uptake in these groups. As the Health Monitor used the EWP, people without a number listed would have been omitted from the sampling frame. This is relevant considering that mobile-only households have increased in South Australia from 5.2% in 2006 to 27.6% in 2013; while national estimates suggest that in 2016, 31% of Australian adults were mobile-only.[62, 63] The use of EWP increases the risk of non-coverage bias and may limit generalisation of findings to the wider South Australian population, with mobile-only households reported to be more likely to contain younger people, unemployed people, renters, and be of lower socio-economic status. [64] However, there is also evidence that when taking into account living arrangements ‘parents and children’ and ‘single parent’ households comprise lower proportions of mobile only households, 22% and 33% respectively, compared to people living in shared households (54%), boarders (41%) and living alone (37%), suggesting parents may be less likely to be omitted compared to other populations. [65] Possible confounders such as a child or parent history of a SRMC and vaccination status, ages and number of children in the household were also not collected. While this is a limitation, the purpose of this study was not to determine associations for specific groups but overall awareness within the ‘parent’ community. This is important given parents are influenced by social interactions, values, beliefs and comparison to others when considering childhood vaccinations. [66] Thus, although healthcare provider messages are key, vaccine decision making happens in conjunction with the indirect messaging from those around you, thereby adding another layer of vaccine messaging within the community.

The study also did not identify Aboriginal and Torres Strait Islander People which is important given Aboriginal and Torres Strait Islander children are recommended to receive the vaccine from 6-months of age and Aboriginal and Torres Strait Islander people of all ages are currently funded on the NIP. This area requires further study, although high coverage (60%) has been identified in the Northern Territory. [14] Even though parents were only from South Australia, limited information exists in Australia on parental awareness of influenza vaccination recommendations for children in the community and patterns of immunisation service use. Lessons learnt from this study could be applied to other jurisdictions around Australia, given the current models for influenza vaccine delivery are similar.

## Conclusions

Parents display low awareness of influenza vaccination recommendations for children, with lower awareness based on place of residence, country of birth, workforce engagement and education level. Developing targeted communication strategies and resources and comprehensive media advertising could help to establish broader community awareness of recommendations for children aged < 5 years and children of all ages with SRMCs. Improving HCP knowledge of recommendations in rural communities as well as equitable assess to vaccines could improve influenza vaccine uptake in these regions. Health care professionals, particularly GPs and paediatric specialists should be encouraged to discuss influenza vaccine with parents at every opportunity.

## Supporting information

S1 Table Multivariable results for the effect of characteristics on awareness of the influenza vaccine recommendation for children aged < 5 years crude versus each additional block of variables (N = 539). (PDF)

S2 Table Multivariable results for the effect of characteristics on awareness of the influenza vaccine recommendation for children with SRMC crude versus each additional block of variables (N = 539). (PDF)

S3 Table Participants' vaccination beliefs by level of concern towards vaccination (N = 539). (PDF)

S4 Table Reason for choice of children's immunisation provider (N = 530). (PDF)

S1 Fig Number of responses to vaccination belief questions that indicate views opposing vaccination/hesitancy by parent reported level of concern towards vaccination in general (N = 539). (PDF)

S2 Fig Source of information on location of immunisation services (N = 530). (PDF)

S1 File Health Monitor questions 2016. (PDF)
