# Supplementary material for: Disparities in parental awareness of children’s seasonal influenza vaccination recommendations and influencers of vaccination
Source: PLoS One. 2020 Apr 9;15(4):e0230425. doi: 10.1371/journal.pone.0230425 (PMC7145195; doi:10.1371/journal.pone.0230425)
Supplement: S1 Table — (PDF) [file pone.0230425.s001.pdf]

S1 Table. Multivariable results for the effect of characteristics on awareness of the influenza vaccine recommendation for <u>children aged < 5 years</u> crude versus each additional block of variables (N=539)

Characteristic		Level	Number of parents	crude			model 1			model 2			model 3			Adjusted Model all covariates		
				OR	95 % CI	p value	OR	95 % CI	p value	OR	95 % CI	p value	OR	95 % CI	p value	OR	95 % CI	p value
Demographic	Age (yrs)	-	539	0.99	(0.94-1.03)	0.562	0.99	(0.95-1.03)	0.749	0.99	(0.95-1.04)	0.699	1.00	(0.96-1.05)	0.985	0.99	(0.94-1.05)	0.787
	Gender	Female	288	0.98	(0.48-2.01)	0.957	1.35	(0.59-1.03)	0.481	1.33	(0.57-3.08)	0.505	1.49	(0.62-3.58)	0.369	1.00	(0.38-2.62)	0.998
	Residence location	Metropolitan (vs Regional)	418	2.03	(1.01-4.10)	0.047	2.33	(1.08-1.03)	0.032	2.35	(1.08-5.13)	0.031	2.51	(1.12-5.62)	0.025	2.91	(1.19-7.09)	0.019
	Country of birth	Australia	436	ref	-	-	ref	-	-	ref	-	-	ref	-	-	ref	-	-
		UK/Ireland	33	0.27	(0.07-1.10)	0.067	0.37	(0.10-1.03)	0.135	0.39	(0.10-1.59)	0.191	0.38	(0.08-1.73)	0.210	0.19	(0.04-0.85)	0.030
		Other	70	1.36	(0.47-3.91)	0.566	1.34	(0.43-1.03)	0.612	1.41	(0.45-4.45)	0.558	1.23	(0.38-4.03)	0.732	0.48	(0.12-1.99)	0.313
	Household speaking language	Non-English (vs English)	44	1.64	(0.39-7.00)	0.502	1.01	(0.18-1.03)	0.994	1.06	(0.18-6.12)	0.949	1.26	(0.20-8.06)	0.807	2.83	(0.48-16.61)	0.250
	Highest educational level	High school or less	148	ref	-	-	ref	-	-	ref	-	-	ref	-	-	ref	-	-
		Trade Certificate	173	0.29	(0.12-0.73)	0.008	0.28	(0.11-1.03)	0.005	0.29	(0.11-0.71)	0.007	0.30	(0.12-0.76)	0.011	0.25	(0.09-0.71)	0.010
		Bachelor or higher	219	0.64	(0.26-1.55)	0.321	0.49	(0.21-1.03)	0.106	0.49	(0.21-1.16)	0.105	0.51	(0.21-1.22)	0.128	0.55	(0.21-1.45)	0.230
	Employment type	Full time	292	ref	-	-	ref	-	-	ref	-	-	ref	-	-	ref	-	-
		Part time/casual	166	1.02	(0.45-2.31)	0.958	0.84	(0.35-1.03)	0.685	0.87	(0.35-2.11)	0.750	0.79	(0.32-1.95)	0.613	0.63	(0.26-1.56)	0.319
		Not working	82	0.16	(0.06-0.42)	<0.001	0.12	(0.04-1.03)	<0.001	0.11	(0.03-0.38)	<0.001	0.09	(0.02-0.33)	<0.001	0.13	(0.04-0.47)	0.002
Parental attitudes to immunisation	Vaccines are necessary to protect my children	Disagree*	9	ref	-	-				ref	-	-	ref	-	-	ref	-	-
		Neutral	29	0.56	(0.07-4.51)	0.588				0.79	(0.07-9.40)	0.849	1.42	(0.12-17.25)	0.785	1.00	(0.11-9.14)	0.997
		Agree**	502	0.45	(0.13-1.57)	0.211				0.37	(0.07-1.98)	0.244	0.39	(0.07-2.21)	0.288	0.51	(0.14-1.85)	0.305
	Belief that "immunisation is important to my everyday life"	No/ low importance#	15	ref	-	-				ref	-	-	ref	-	-	ref	-	-
		Neutral	13	0.35	(0.03-3.75)	0.388				1.03	(0.05-23.14)	0.984	0.50	(0.01-18.53)	0.709	1.62	(0.08-31.57)	0.749
		Important##	511	0.58	(0.09-3.82)	0.568				1.43	(0.13-16.31)	0.771	0.82	(0.06-11.44)	0.882	1.10	(0.13-9.45)	0.930
Health service use	Immunisation service provider	GP	355	ref	-	-							ref	-	-	ref	-	-
		Community clinic	44	0.29	(0.09-0.94)	0.040							0.24	(0.07-0.87)	0.030	0.27	(0.05-1.48)	0.132
		Child health clinic	23	0.96	(0.19-4.92)	0.960							0.88	(0.20-3.99)	0.873	0.47	(0.09-2.61)	0.389
		Combination†	85	0.70	(0.26-1.86)	0.471							0.58	(0.22-1.51)	0.263	0.44	(0.18-1.06)	0.067
		Other††	23	0.53	(0.10-2.78)	0.450							0.51	(0.13-1.98)	0.331	0.19	(0.05-0.80)	0.024
		Don't vaccinate	9	1.29	(0.21-8.08)	0.782							0.32	(0.03-3.91)	0.375	0.92	(0.12-7.05)	0.933
	Youngest child has SRMC	Yes	26	0.48	(0.13-1.77)	0.271							1.23	(0.33-4.61)	0.763	0.95	(0.20-4.59)	0.949
Aware of influenza recommendation for children with SRMC		Yes	277	6.421	(2.73-15.11)	<0.001										10.46	(4.44-24.63)	<0.001

Footnote: SRMC: Special Risk Medical Conditions; Disagree* included disagree/ strongly disagree; Agree** included agree/ strongly agree; No/ low importance# included responses 'Not at all/ somewhat important'; ## included Important/ Very important; † included a combination of providers (from MP or clinics); ††other were school (n= 9), hospital (n=4), chemist (n=4), Aboriginal Health Service (n=4) and 'Could not recall' (n=2).
