# Supplementary material for: Disparities in parental awareness of children’s seasonal influenza vaccination recommendations and influencers of vaccination
Source: PLoS One. 2020 Apr 9;15(4):e0230425. doi: 10.1371/journal.pone.0230425 (PMC7145195; doi:10.1371/journal.pone.0230425)
Supplement: S2 Table — (PDF) [file pone.0230425.s002.pdf]

S2 Table. Multivariable results for the effect of characteristics on awareness of the influenza vaccine recommendation for children with SRMC crude versus each additional block of variables (N=539)

Characteristic		Level	Number of parents	crude			model 1			model 2			model 3			Adjusted Model all covariates		
				OR	95 % CI	p value	OR	95 % CI	p value	OR	95 % CI	p value	OR	95 % CI	p value	OR	95 % CI	p value
Demographic	Age (yrs)	-	539	1.01	(0.97-1.05)	0.513	1.02	(0.98-1.05)	0.398	1.02	(0.98-1.05)	0.388	1.01	(0.97-1.05)	0.580	1.02	(0.97-1.06)	0.460
	Gender	Female	288	1.78	(0.95-3.34)	0.071	2.22	(1.02-4.84)	0.045	2.15	(0.99-4.68)	0.053	2.39	(1.09-5.25)	0.030	2.47	(0.97-6.31)	0.058
	Residence location	Metropolitan (vs Regional)	418	1.12	(0.60-2.10)	0.712	1.14	(0.57-2.27)	0.713	1.14	(0.57-2.28)	0.720	1.14	(0.54-2.39)	0.735	0.77	(0.34-1.76)	0.535
	Country of birth	Australia	436	ref	-	-	ref	-	-	ref	-	-	ref	-	-	ref	-	-
		UK/Ireland	33	3.72	(1.03-13.48)	0.046	4.06	(0.98-16.81)	0.053	4.34	(1.05-17.97)	0.043	4.76	(1.15-19.64)	0.031	7.63	(1.86-31.31)	0.005
		Other	70	1.48	(0.51-4.28)	0.469	3.43	(1.06-11.10)	0.040	3.41	(1.05-11.05)	0.041	3.77	(1.15-12.41)	0.029	3.93	(0.94-16.42)	0.060
	Household speaking language	Non-English (vs English)	44	0.85	(0.20-3.57)	0.827	0.38	(0.06-2.20)	0.279	0.42	(0.07-2.52)	0.342	0.31	(0.05-2.03)	0.224	0.28	(0.04-1.89)	0.190
	Highest educational level	High school or less	148	ref	-	-	ref	-	-	ref	-	-	ref	-	-	ref	-	-
		Trade Certificate	173	0.88	(0.38-2.03)	0.770	0.82	(0.36-1.86)	0.628	0.87	(0.37-2.01)	0.738	0.97	(0.43-2.19)	0.937	1.64	(0.69-3.89)	0.266
		Bachelor or higher	219	0.77	(0.33-1.77)	0.535	0.62	(0.26-1.44)	0.265	0.63	(0.27-1.48)	0.291	0.69	(0.30-1.63)	0.402	0.89	(0.36-2.19)	0.808
	Employment type	Full time	292	ref	-	-	ref	-	-	ref	-	-	ref	-	-	ref	-	-
		Part time/casual	166	1.65	(0.82-3.34)	0.162	1.07	(0.48-2.38)	0.872	1.13	(0.51-2.54)	0.762	1.22	(0.56-2.68)	0.613	1.37	(0.59-3.19)	0.460
		Not working	82	0.89	(0.36-2.18)	0.797	0.47	(0.17-1.31)	0.149	0.48	(0.17-1.36)	0.168	0.41	(0.16-1.07)	0.068	0.85	(0.31-2.33)	0.748
Parental attitudes to immunisation	Vaccines are necessary to protect my children	Disagree*	9	ref	-	-				ref	-	-	ref	-	-	ref	-	-
		Neutral	29	1.40	(0.14-13.98)	0.775				0.60	(0.04-8.00)	0.697	1.22	(0.07-21.83)	0.894	1.13	(0.12-10.88)	0.917
		Agree**	502	0.92	(0.27-3.16)	0.895				0.52	(0.08-3.58)	0.506	0.47	(0.06-3.45)	0.457	0.63	(0.15-2.66)	0.531
	Belief that "immunisation is important to my everyday life"	No/ low importance#	15	ref	-	-				ref	-	-	ref	-	-	ref	-	-
		Neutral	13	0.71	(0.07-7.52)	0.773				0.83	(0.03-27.00)	0.918	0.21	(0.00-9.18)	0.420	0.15	(0.01-2.30)	0.173
		Important##	511	1.28	(0.19-8.40)	0.798				1.98	(0.08-46.73)	0.673	0.84	(0.03-20.55)	0.917	0.88	(0.09-8.77)	0.912
Health service use	Immunisation service provider	GP	355	ref	-	-							ref	-	-	ref	-	-
		Community clinic	44	0.65	(0.24-1.79)	0.408							0.51	(0.14-1.85)	0.307	0.85	(0.18-4.10)	0.843
		Child health clinic	23	2.37	(0.66-8.56)	0.187							2.28	(0.59-8.81)	0.233	2.55	(0.77-8.48)	0.128
		Combination†	85	1.20	(0.52-2.78)	0.669							1.14	(0.46-2.82)	0.777	1.48	(0.62-3.51)	0.373
		Other††	23	4.32	(1.24-15.06)	0.022							7.60	(1.95-29.56)	0.003	13.40	(2.93-61.23)	0.001
		Don't vaccinate	9	0.76	(0.12-4.67)	0.763							0.19	(0.01-3.67)	0.268	0.23	(0.02-2.23)	0.203
	Youngest child has SRMC	Yes	26	0.92	(0.26-3.27)	0.899							0.85	(0.22-3.31)	0.810	0.74	(0.15-3.65)	0.713
Aware of influenza recommendation for children <5 years		Yes	177	6.420956	(2.73-15.11)	<0.001										10.21735	(4.39-23.77)	<0.001

Footnote: SRMC: Special Risk Medical Conditions; Disagree* included disagree/ strongly disagree; Agree** included agree/ strongly agree; No/ low importance# included responses 'Not at all/ somewhat important'; ## included Important/ Very important; † included a combination of providers (from MP or clinics); ††other were school (n= 9), hospital (n=4), chemist (n=4), Aboriginal Health Service (n=4) and 'Could not recall' (n=2).
