# Supplementary material for: Disparities in parental awareness of children’s seasonal influenza vaccination recommendations and influencers of vaccination
Source: PLoS One. 2020 Apr 9;15(4):e0230425. doi: 10.1371/journal.pone.0230425 (PMC7145195; doi:10.1371/journal.pone.0230425)
Supplement: S3 Table — (PDF) [file pone.0230425.s003.pdf]

S4 Table. Participants' vaccination beliefs by level of concern towards vaccination (N= 539)

Vaccination belief	Level of concern	Agree/ strongly agree		Neither disagree/ agree		Disagree/ strongly disagree	
		n	(%) 95% CI	n	(%) 95% CI	n	(%) 95% CI
**Immunisation is important to my everyday life#**	**Overall**	**511**	**94.8 (90.8-97.1)**	**15**	**2.7 (1.3-5.8)**	**13**	**2.5 (1.0-6.0)**
	No or minor concern*	485	97.5 (94.3-98.9)	13	2.5 (1.1-5.7)	0	0.0 (-)
	High level of concern*	16	72.2 (30.2-94.0)	0	0.0 (-)	6	27.8 (6.0-69.8)
	Delay or exclude vaccines	10	52.1 (23.5-79.4)	2	10.9 (1.5-49.2)	7	37.0 (13.2-69.4)
**Vaccines are necessary to protect my children**	**Overall**	**502**	**93.1 (88.8-95.8)**	**9**	**1.6 (0.6-3.9)**	**29**	**5.3 (2.9-9.4)**
	No or minor concern*	481	96.6 (93.1-98.3)	2	0.3 (0.1-1.5)	15	3.1 (1.4-6.6)
	High level of concern*	13	59.3 (21.9-88.4)	3	12.9 (1.7-56.2)	6	27.8 (6.0-69.8)
	Delay or exclude vaccines	8	41.7 (16.9-71.6)	4	21.3 (6.2-52.6)	7	37.0 (13.2-69.4)
**As other children are vaccinated, it isn't necessary to vaccinate my children**	**Overall**	**65**	**12.1 (8.0-17.8)**	**7**	**1.3 (0.5-3.4)**	**467**	**86.6 (80.8-90.8)**
	No or minor concern*	54	10.9 (6.9-16.9)	3	0.6 (0.1-2.7)	441	88.4 (82.4-92.6)
	High level of concern*	2	11.4 (2.1-43.9)	1	5.2 (0.6-32.1)	18	83.3 (51.1-96.0)
	Delay or exclude vaccines	8	42.8 (17.2-72.9)	3	14.8 (3.1-48.3)	8	42.4 (17.2-72.3)
**Serious side effects are too common for me to accept**	**Overall**	**46**	**8.5 (5.4-13.0)**	**14**	**2.5 (1.3-4.9)**	**480**	**89.0 (84.2-92.4)**
	No or minor concern*	28	5.6 (3.1-10.0)	12	2.4 (1.2-5.0)	458	92.0 (87.4-95.0)
	High level of concern*	6	26.7 (6.9-64.2)	1	2.8 (0.3-19.9)	15	70.5 (33.7-91.8)
	Delay or exclude vaccines	12	61.2 (31.0-84.7)	1	5.1 (0.7-30.0)	7	33.7 (12.1-65.1)
**Vaccination services for my children are difficult to access**	**Overall**	**17**	**3.1 (1.5-6.4)**	**9**	**1.6 (0.6-4.4)**	**514**	**95.3 (91.6-97.4)**
	No or minor concern*	12	2.5 (1.0-6.1)	6	1.2 (0.3-3.8)	480	96.4 (92.6-98.3)
	High level of concern*	1	2.8 (0.3-19.9)	3	12.9 (1.7-56.2)	18	84.3 (44.7-97.3)
	Delay or exclude vaccines	4	19.9 (5.6-51.2)	0	0.0 (-)	16	80.1 (48.8-94.4)

Footnote: * child receives all vaccines; # Participants were asked to rate the importance of immunisation to their everyday life on a Likert scale however responses were important/very important (agree/strongly agree), neutral and not at all or somewhat important (disagree/strongly disagree).
