# Supplementary material for: Disparities in parental awareness of children’s seasonal influenza vaccination recommendations and influencers of vaccination
Source: PLoS One. 2020 Apr 9;15(4):e0230425. doi: 10.1371/journal.pone.0230425 (PMC7145195; doi:10.1371/journal.pone.0230425)
Supplement: S4 Table — (PDF) [file pone.0230425.s004.pdf]

S5 Table. ***Reason for Choice of Children's Immunisation Provider (N=530)***

Reason for Choice of Immunisation Provider	n	% (95% CI)
Close to my home/easy to get to	251	47.4 (39.6 -55.3)
They have our medical records	161	30.3 (23.5 -38.1)
Trustworthy	136	25.7 (18.6 -34.4)
Medical doctor provides the service	86	16.2 (11.6 -22.1)
Can make an appointment	34	6.4 (4.0 -10.1)
No waiting times	31	5.8 (2.9 -11.3)
Opening hours	18	3.5 (1.4 -8.5)
Free of charge	17	3.3 (1.4 -7.2)
Other††	12	2.3 (1.0 -5.1)
Unaware of other options	12	2.3 (1.1 -4.8)
Only option- rural	10	1.9 (0.9 -3.9)
Staff rapport	10	1.9 (0.7 -5.2)
Vaccine availability	7	1.4 (0.5 -3.6)
No sick people in waiting area	3	0.6 (0.1 -2.6)
Experienced staff/technique	2	0.4 (0.1 -1.4)
Don't know	2	0.4 (0.0 -2.5)

Footnote: Multiple response. These data excluded parents who did not vaccinate (n=9; weighted data); ††: other reasons were 'as listed in blue (baby) book' n=1, 'depends on the situation' n=1, 'word of mouth' n=3, child not yet vaccinated n=2, told to go there n=2 and other health service provided there n=3.
