# Supplementary material for: Disparities in parental awareness of children’s seasonal influenza vaccination recommendations and influencers of vaccination
Source: PLoS One. 2020 Apr 9;15(4):e0230425. doi: 10.1371/journal.pone.0230425 (PMC7145195; doi:10.1371/journal.pone.0230425)
Supplement: S1 Fig — (PDF) [file pone.0230425.s005.pdf]

S3 Fig. Number of responses to vaccination belief questions that indicate views and beliefs opposing vaccination by parent reported level of concern towards vaccination in general (N=539)

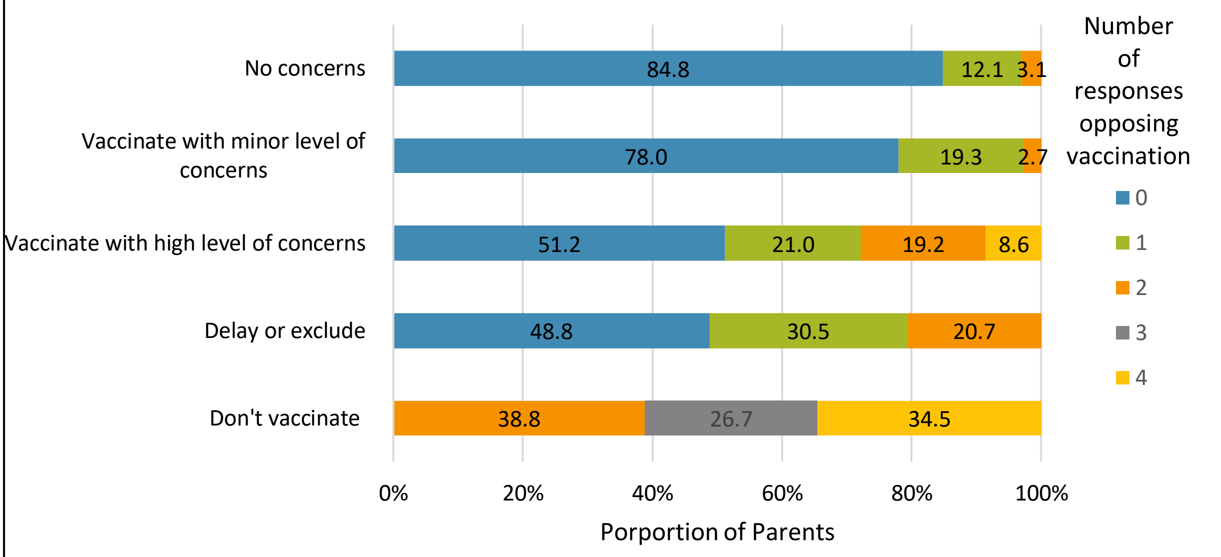

Footnote: Questions included, 'How important do you think immunisation is to your everyday life?'; ' Vaccines are necessary to protect my child/children'; 'Because other children are vaccinated, it isn't necessary to have my child/children vaccinated'; 'Serious side effects are too common for me to accept'
