# Supplementary material for: Disparities in parental awareness of children’s seasonal influenza vaccination recommendations and influencers of vaccination
Source: PLoS One. 2020 Apr 9;15(4):e0230425. doi: 10.1371/journal.pone.0230425 (PMC7145195; doi:10.1371/journal.pone.0230425)
Supplement: S2 Fig — (PDF) [file pone.0230425.s006.pdf]

S6 *Fig. Source of information on location of Immunisation Services (N=530)*

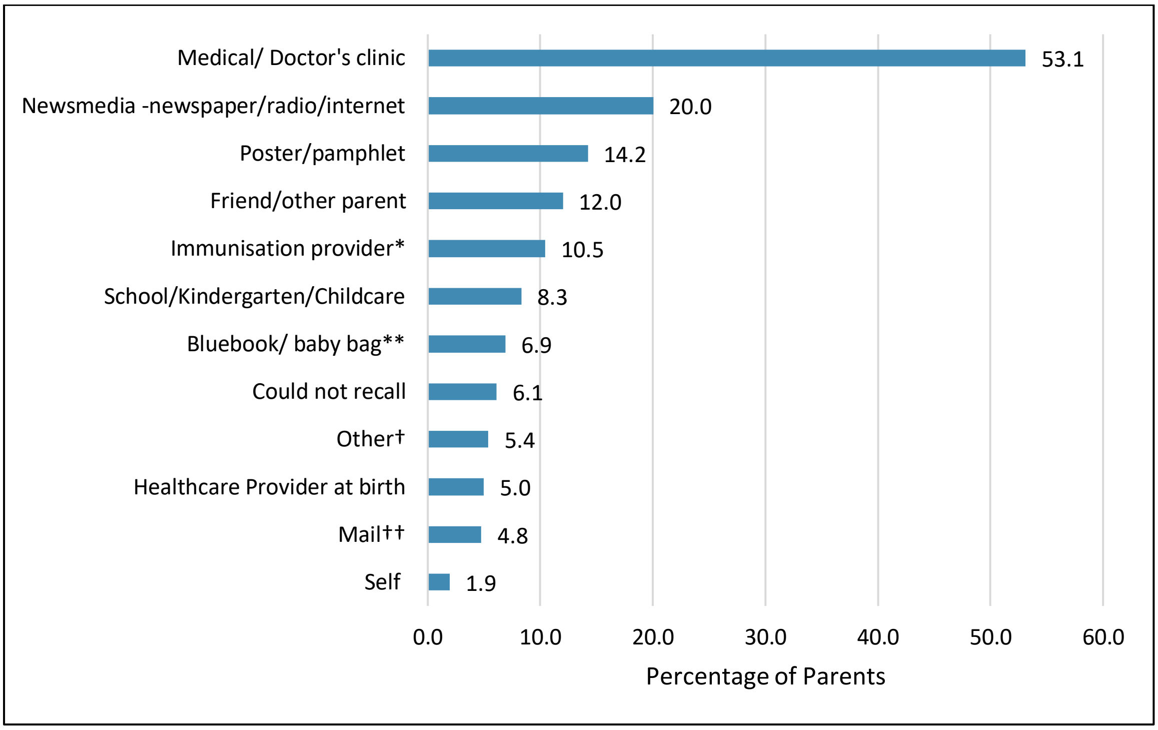

Footnote: Multiple response. *Community Immunisation Clinic, Child Health Clinic or hospital; ** Bluebook is a record book given to new parents at birth to record important health information and milestones; the baby bag is provided to parents at their first antenatal appointment and again at the child's birth; † other included work n=5, Centrelink n=17, email n=4, pharmacy/ drug store n=1, don't receive n=1; †† A number of parents reported the source of information as 'mail' (postal) however, as no further explanation was provided, we assume (but cannot be certain) that parents were referring to information received from Medicare after the baby was registered.
