# Supplementary material for: Disparities in parental awareness of children’s seasonal influenza vaccination recommendations and influencers of vaccination
Source: PLoS One. 2020 Apr 9;15(4):e0230425. doi: 10.1371/journal.pone.0230425 (PMC7145195; doi:10.1371/journal.pone.0230425)
Supplement: S1 File — (PDF) [file pone.0230425.s007.pdf]

S7. Health Monitor Questions 2016

Demographic variables

A.1	How old are you?
A.2	Which age group are you in? Would it be…
A.3	Sex
A.4	Including yourself how many people aged 18 or over live in this household?
A.5	What is the Postcode of the house?
A.6	What town or suburb do you live in?
Z.1	Which of the following best describes your current marital status?
Z.2	What is your work status?
Z.3	Do you receive any of the following pension benefits?
Z.4	In which country were you born?
Z.5	What year did you arrive in Australia?
Z.6	Are you of Aboriginal or Torres Strait Islander origin?
Z.7	What is the main language you speak at home?
Z.8	Which best describes the highest educational qualification you have obtained?
Z.9	The next question is about housing. Is this dwelling…
Z.10	Household income…

Immunisation variables

B.4	On a scale of 1-5 how important do you think immunisation is to your everyday life?
B.16	Did you know that children with high risk medical conditions are recommended to receive a free flu vaccine each year?
B.17	Does your child have any of these high-risk medical conditions?
B.18	Did your child with a medical condition receive a flu vaccine this year?
B.19	Did you know that all children from 6 months of age to less than 5 years are recommended to receive the seasonal influenza vaccine?
B.20	What would be the most influential for you in deciding to have your child receive a flu vaccine?
B.21	Vaccines are necessary to protect my child/children (Likert scale)
B.22	Because other children are vaccinated, it isn't necessary to have my child/children vaccinated (Likert scale)
B.23	Serious side effects are too common for me to accept (Likert scale)
B.24	It is difficult to access vaccination services for my child/children (Likert scale)
B.25	Which of the following best describes your beliefs about vaccination?
B.26	Where do you choose to go to have your child vaccinated?
B.27	Do you have any difficulties in getting there?
B.28	What are some of those difficulties?
B.29	Why do you choose to go there over other clinics?
B.30	Do you think there is sufficient information in the community about where you can go to get vaccinated?
B.31	Where do you receive your information on where to go to get vaccinated?
B.32	How would you rate your experience of the most recent vaccination service your child received? (Likert scale)
B.33	Why was your recent vaccination experience poor?
B.34	Although vaccines provided under the National Immunisation Program are free, did you have to pay for the service at your most recent vaccination?
B.35	Adults are now allowed to get their flu vaccine from a pharmacy. If a pharmacist was allowed to vaccinate your child, would you take them there?
